# Knowledge and Beliefs about Clinical Trials among Adults in Poland: A Cross-Sectional Study

**DOI:** 10.3390/clinpract14040104

**Published:** 2024-07-02

**Authors:** Natalia Cięszczyk, Marcin Czech, Łukasz Pronicki, Mariusz Gujski

**Affiliations:** 1 Department of Public Health, Medical University of Warsaw, 02-091 Warszawa, Poland; 2 Institute of Mother and Child, 01-211 Warszawa, Poland

**Keywords:** clinical trials, awareness, knowledge

## Abstract

Clinical trials, by contributing to the development of diagnostics and to the search for modern, more effective, and safer therapies, have become one of the most important elements of the healthcare system. They enable the introduction of innovative drugs and treatments that can significantly improve patients’ quality of life. Not only does this research help to understand disease mechanisms, but it also enables the personalization of therapy, which often increases the effectiveness of treatment. Public awareness of clinical trials helps build trust in science and medicine, which is fundamental to the effective functioning of the healthcare system. The aim of this study was to assess Poles’ knowledge and beliefs about clinical trials. Methods: The survey was conducted among Poles aged 18 and over with the help of an external company, Ariadna, which is an independent research panel. The questionnaire contained 22 questions, of which 13 questions concerned beliefs and attitudes towards clinical trials. Results: One thousand and seventy-nine participants took part in the study (*n* = 1079). The mean age of respondents was 44.96 years (SD = 16.30). Slightly more women (*n* = 568, 52.6%) than men (*n* = 511, 47.4%) took part in the study. Among the respondents, 86.5% (*n* = 933) were aware of clinical trials. The main sources of information about clinical trials were the media (53.8%) including the Internet (*n* = 355, 32.9%), TV (*n* = 175, 16.2%), press (*n* = 30, 2.8%), and radio (*n* = 21, 1.9%). 43.2% (*n* = 466) of respondents reported little knowledge of clinical trials, while more than three quarters (*n* = 805, 75.2%) said they would like to learn more about clinical trials. Most respondents (*n* = 879, 81.4%) agreed with the statement that participation in a clinical trial is completely voluntary, and more than half (*n* = 580, 53.7%) agreed with the statement that hospitals participating in clinical trials provide better healthcare. The statement that the results of clinical trials are made available to the public was disagreed with by 37.2% (*n* = 402) of participants. Only 30.3% (*n* = 327) of participants agreed that clinical trials should be conducted with children. Most respondents (*n* = 638, 59.1%) agreed with the statement that a patient in a clinical trial is insured. 48.3% (*n* = 521) of participants are aware that a clinical trial can be withdrawn from at any time. Conclusions: Poles rate their knowledge of clinical trials as low and would like to learn more. Poles’ knowledge of clinical trials is mainly based on commercial sources.

## 1. Introduction

Over the past 25 years, Poland has strengthened its position as a significant player in the global market for innovative biopharmaceutical commercial clinical trials. In 2019, Poland ranked 11th globally in terms of market share in the innovative biopharmaceutical commercial clinical trials market. From 2014 to 2019, Poland experienced one of the most dynamic growth rates in this market share globally, allowing Poland to rank 5th, behind only China, Spain, South Korea, and Taiwan [1]. In Poland, the number of registered clinical trials has remained stable at more than 400 trials per year for the last few years. In 2014, the President of the Office for Registration of Medicinal Products, Medical Devices, and Biocidal Products registered 396 trials [2]. In 2016, there were already 458 [3], in 2019—603 [4], and in 2022—688 applications to start a clinical trial of a medicinal product. For the next consecutive year, this was the highest number of applications to start a clinical trial submitted in a calendar year in the authority’s history. Of the applications submitted, 52 were for non-commercial trials (compared to 41 such applications in 2021). Phase III (approximately 51%) and Phase II (approximately 30%) clinical trials predominate among the applications submitted in 2022. [5]. In 2020, 25,000 patients in Poland benefited from innovative therapies in clinical trials. With a trial availability of 63% compared to the USA—our country ranks among the countries with a high level of clinical trial availability to patients (12th in the world and 8th in Europe, ahead of Germany, France, Italy, and the UK, among others) [1]. In 2022, more than 20% of registered clinical trials are in oncology. The other main fields of medicine under which clinical trials are registered in Poland are neurology (12%), dermatology (10%), cardiology (8%), and pulmonology (6%) [6]. Clinical trials are an intrinsic component of today’s medicine, and their conduct has a key impact on increasing therapeutic efficacy, which translates into the health and well-being of the general population. Although they undeniably contribute to the positive development of the health sector, they often face an unfavorable reception from the public [7]. Awareness of clinical trials remains insufficient, resulting in many patients missing out on the opportunity to benefit from the latest medicines that can not only save or prolong life but also significantly improve the quality of life. Negative associations and a lack of sufficient awareness of the nature of these trials are significant obstacles to their further development [8]. There are many factors that influence this perception of clinical trials. Many people do not realize that they can take part in this type of research, and they do not understand what it is and what it means [9]. Therefore, this study aims to assess the knowledge and beliefs about clinical trials among Polish adults.

## 2. Materials and Methods

### 2.1. Study Design

The survey was conducted on a nationwide sample of Poles. We chose the computer-assisted web interviewing (CAWI) method because of its efficiency and ability to reach a wide range of respondents. We created a questionnaire, which was entrusted to Ariadna, an independent national research panel for implementation. Ariadna provides researchers with the ability to conduct nationwide research, including surveys and experiments, to the highest standards of accuracy and reliability. During the research, the Ariadna panel was the holder of a current certificate of the Interviewer Quality Control Programme, which is evidence of the high quality of the research services offered and is issued based on annual independent audits conducted by the Polish Association of Researchers in Public Opinion and Marketing [10]. Survey participants are invited to complete the survey via a personalised link sent to their email address registered in the Ariadna panel. The survey is completed by individuals with a confirmed identity. Upon completion of the survey, Ariadna provides an anonymised set of data in the form of statistical summaries. Ariadna operates based on the Personal Data Protection Act and the ICC/ESOMAR Code of Ethics, created in cooperation between the European Association of Opinion and Market Researchers and the International Chamber of Commerce in Poland [11]. The data collected were then statistically analysed.

### 2.2. Ethical Aspects

The study was commenced after receiving approval from the Bioethics Committee. On 16 January 2023, by decision number AKBE/2/2023, the research project together with the questionnaire received approval from the Ethics Committee of the Warsaw Medical University. The data collected as part of the survey were fully anonymous, making it impossible to identify individual study participants. All procedures related to the study, with the participation of the participants, were carried out in accordance with the ethical standards set by the relevant institutions or national research regulators, and in accordance with the principles of the Declaration of Helsinki.

### 2.3. Participants

The survey was conducted between January and February 2023, covering a national sample of Polish residents aged 18 or over. To ensure that the sample was representative, its structure in terms of gender, age, and place of residence was adapted to the distribution of these characteristics in the adult Polish population. During the registration process, the identity of each participant was verified, with full anonymity and protection of personal data ensured.

### 2.4. Questionnaire Development

At the outset, the goals of the survey were defined and what information in the questionnaire is needed to achieve those goals. The research tool was a nationwide self-completion questionnaire developed for this study. In preparing the questionnaire, we analysed previously published studies on perceptions of clinical trials among different populations. The survey questionnaire was developed based on publicly available questions used in a study of public awareness and attitudes toward clinical trials in India [12,13] and Jordan [14]. The questions were translated into Polish, consulted with experts, and adapted to the context of Polish society. The questions were arranged in a logical and coherent manner so that they flowed smoothly from the general to the specific. The questionnaire was divided into sections covering areas of knowledge and attitudes toward clinical research. The questionnaire contained 22 questions, of which 13 questions related to beliefs and attitudes towards clinical trials. Additional questions also asked for background information, including sex, size of residence, education, province, and marital status. The questionnaire was tested on a limited group of people (*n* = 43) not related to the medical industry prior to the study.

Awareness of clinical trials was assessed using the question “Have you ever heard of clinical trials?” (“Yes”/“No”). An additional question on the source of knowledge about clinical trials was addressed to all respondents who declared knowledge of clinical trials. All 8 questions were designed to assess attitudes and beliefs towards clinical trials. The questions had a 5-point response scale of: 1 = “Definitely yes”, 2 = “Rather yes”, 3 = “Probably not”, 4 = “Definitely not” and 5 = “Hard to say”.

### 2.5. Statistical Analysis

Continuous variables were condensed by calculating their average and standard deviation. Furthermore, we provided information on the median, interquartile range, range, and kurtosis. For nominal variables, we employed counts and percentages for summarization purposes. To analyse correlations, Spearman’s ρ was utilized, and the significance of these correlations was assessed using a *p*-value based on Hollander and Wolfe’s method. To account for multiple comparisons, we applied a Benjamini–Hochberg correction to the *p*-values. A relationship was deemed significant when the *p*-value was less than 0.05. The analysis was carried out in the R language environment. No normal distribution (or any other) was assumed in the structure of the responses due to the lack of data to make this assumption. Testing for normal distribution may be troublesome in such cases (see e.g., F.E. Harrell Regression Modeling Strategies for further references). Tests used in the analysis are non-parametric and do not need normal distribution to be effective. A summary of the methods is presented in Table 1.

## 3. Results

A total of 7330 invitations were sent to complete the survey. Data were obtained from 1079 respondents, including 568 women (52.6%) and 511 men (47.4%). The response rate was 26.04% and 1909 records were excluded due to missing data. The mean age of respondents was 44.96 years (SD = 16.30) with no age differences between men and women. Of the 1079 participants, 404 (37.4%) lived in the countryside, 134 (12.4%) lived in a large city of more than 500,000 inhabitants, and 140 (13%) lived in a small town of up to 20,000 inhabitants. Only 96 people (8.9%) lived in a medium-sized city with 20,001–50,000 inhabitants and 87 people (8.1%) lived in a large city with 100,001–200,000 inhabitants.

Among the respondents, 155 (14.4%) came from the Mazowieckie Voivodeship and 136 (12.6%) from the Śląskie Voivodeship. The fewest respondents came from the Opolskie Voivodeship—24 participants (2.2%). Most participants—383 (35.5%)—had a master’s degree or secondary education—338 (31.3%). 111 participants (10.3%) had post-secondary education and 111 participants (10.3%) had basic education. Ninety-six participants (8.9%) had completed a bachelor’s degree and only 40 participants (3.7%) had primary education or had graduated from middle school. The dominant group of participants (*n* = 575, 53.3%) declared that they were in a relationship. A total of 333 participants (30.9%) were single, 116 (10.8%) were divorced and 55 (5.1%) were widowed or widower. Detailed characteristics of the participants are shown in Table 2.

**Figure 1:**
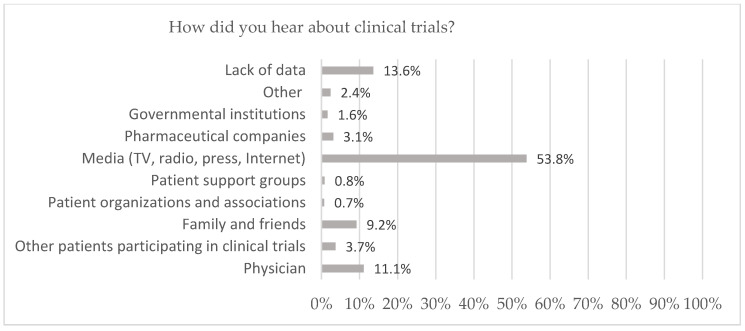
Source of knowledge about clinical trials for adults in Poland.

Among participants who were aware of clinical trials (*n* = 933, 86.5%), the main sources of information about clinical trials were the media (53.8%) including the Internet (*n* = 355, 32.9%), TV (*n* = 175, 16.2%), press (*n* = 30, 2.8%) and radio (*n* = 21, 1.9%). A total of 11.1% (*n* = 120) of respondents indicated that they learned about clinical trials from doctors, and 9.2% (*n* = 99) from family members or friends. A total of 3.7% (*n* = 40) of respondents obtained knowledge about clinical trials from other patients participating in clinical trials and 3.1% (*n* = 33) from pharmaceutical companies. A total of 1.6% (*n* = 17) of respondents answered that they knew about clinical trials from government institutions. The fewest indicated that they acquired their knowledge from patient support groups (*n* = 9, 0.8%) or patient organizations and associations (*n* = 8, 0.7%) (Figure 1).

Among the respondents, the vast majority (*n =* 955, 88.5%) declared that they had never participated in clinical trials and 11.5% (*n =* 124) of participants had taken part in clinical trials (Table 3). 43.2% (*n =* 466) of respondents declared little knowledge of clinical trials and 23.4% (*n =* 252) declared they knew nothing about clinical trials. Only 14.8% (*n =* 160) of respondents rated their knowledge as high and 1.7% (*n =* 18) as very high (Table 3). There were no significant differences between the respondents’ knowledge status and their education. Willingness to learn about clinical trials was declared by most participants (*n =* 805, 75.2%), as shown in Table 3. There were no significant differences in the declaration of willingness to learn about clinical trials between men and women.

Most participants (*n* = 879, 81.4%) agreed (“Rather yes” or “Definitely yes”) that participation in a clinical trial is completely voluntary, and more than half of respondents (*n* = 580, 53.7%) believe that hospitals participating in clinical trials provide better healthcare. 33.9% (*n* = 366) were undecided and could not answer this question. The remainder of the participants (*n* = 133, 12.3%) disagreed with this statement (Table 4). Among participants, 37.2% (*n* = 402) disagreed (“Probably not” or “Definitely not”) with the statement that the results of clinical trials are made available to the public. To the same statement, 34.5% (*n* = 373) of participants answered, “Rather yes” or “Definitely yes”. The remaining 28.2% (*n* = 304) could not answer this question. 48.3% (*n* = 521) of participants are aware that a clinical trial can be withdrawn from at any time. 20.2% (*n* = 218) of respondents believe that you cannot and 31.5% (*n* = 340) are undecided.

Most respondents (*n* = 638, 59.1%) agreed with the statement that a patient in a clinical trial is insured, 11.4% (*n* = 123) of participants think not and 29.5% (*n* = 318) cannot decide. 43.7% (*n* = 471) disagreed (“Probably not” or “Definitely not”) with the statement that all participants in the clinical trial receive the investigational medicinal product, 29.5% (*n* = 318) of participants agreed with the statement and 26.9% (*n* = 290) cannot decide. 35.7% (*n* = 386) of respondents believe that clinical trials involving children should not be conducted. 30.3% (*n* = 327) of participants declared that clinical trials involving children should be conducted and 33.9% (*n* = 366) of participants were undecided. Thus, most participants (*n* = 696, 64.5%) would allow their child to take part in a clinical trial as the only therapeutic option (there is no other treatment), 13.0% (*n* = 140) of participants declare that they would not give such consent and 22.5% (*n* = 243) of people cannot decide.

## 4. Discussion

In our study, almost all respondents (86.5%) had previously heard of clinical trials. Referring to a 2018 article, which also surveyed Poles’ awareness of clinical trials (with 284 respondents), it was shown that at that time 69.9% of respondents were aware of clinical trials [15]. The differences in results between that and the current survey can be understood in the context of the passage of time. It is noteworthy that the mass public exposure to the COVID-19 vaccine development process, including clinical trials related to its efficacy and safety, may have significantly influenced people’s awareness of clinical trials. Since 2018, and especially over the past few years, the COVID-19 pandemic has become a major topic of discussion around the world, leading to increased public attention to research processes, including the clinical trial stage, as a key element in drug and vaccine development. The public’s awareness of clinical trials in Poland may now be more developed and popularized since more and more new clinical trials are being registered in Poland each year, with an increasing number of patients having access to them.

For comparison, in a 2015 article where the level of awareness of clinical trials in the US was studied, awareness of clinical trials increased from 68.0% to 74.0% between 2008 and 2012 [16]. In another study conducted in India, 52.5% of 400 participants surveyed had previously heard of clinical trials [17].

Although Poles’ awareness of the existence of clinical trials is at a high level, respondents’ declared knowledge of clinical trials is low. 43.2% of respondents declared little knowledge about clinical trials, 23.4% of respondents declared that they knew nothing about clinical trials and 17.0% of respondents were undecided. It can be concluded that the undecidedness is also due to a lack of knowledge about clinical trials and therefore respondents found it difficult to express their views on the subject. When a person does not have enough information about an issue, he or she may feel uncertain in deciding. The lack of adequate information makes it difficult to evaluate the different choice options [18]. Deficiencies in the knowledge of clinical research in Polish society may be due to insufficient education about clinical research already in the school system. The education curriculum of future doctors and other medical professions should include the topic of clinical research. Currently, only some universities offer optional seminars for interested students. The consequences of the lack of education at the university level are borne, among others, by patients who rarely receive information from their doctors about clinical trials for which they may be eligible [19].

Many respondents (75.2%) expressed a desire to learn more about clinical trials. Providing information and raising awareness about clinical trials and participation is a challenge for those responsible for planning, organizing, and implementing clinical trials. To reach as many patients as possible, parallel activities on multiple levels can have the best effect. It is very important to organize educational events aimed at patients and their families: lectures, workshops, educational panels, and webinars on clinical trials. The aim of such events should be to promote the possibility of participation, to raise awareness of the importance of clinical trials themselves, and to present possible benefits and risks while avoiding incentives [19].

We observed that only 11.1% of respondents get information about clinical trials from doctors and from patient support groups (0.8%) or patient organizations and associations (0.7%). Most participants learned about clinical trials from the media (53.8%) including the Internet (32.9%) and TV (16.2%). Information obtained from such sources is not scientifically verified and may be biased. Media coverage tends to be very general and portrays clinical trials as dodgy medical experiments in which the participant is exploited by pharmaceutical companies. An example of this is an article on a popular website with the title “You can earn £2500 a hand. We are looking for healthy, adult men to participate in clinical drug trials” [20] or “Hunting guinea pigs” [21]. Similar articles appear in a popular American monthly magazine: “Poor men around the world get a measly pittance for testing drugs on their own bodies” [22].

It is therefore essential to conduct nationwide public campaigns to raise patient awareness of clinical trials and to present participation in clinical trials as an alternative to standard healthcare. Campaigns should promote sound knowledge in a way that is accessible to the audience [19].

An example of this is the campaign run for 3 months in Scotland—the ‘Get Randomized’. The main aim of the campaign was to raise awareness of clinical trials. The campaign results showed that the term ‘randomized clinical trial’ was recognized after the campaign by 37.8% of respondents, compared to 28.6% before the campaign. An increase in respondents’ awareness was also observed with the topic that clinical trials increase access to innovative treatments (2.4% pre-campaign to 8.4% post-campaign) [23].

Our survey found that only 11.1% of respondents learned about clinical trials from a doctor. In a study published in 2021 in the US, respondents (59.0%) identified physicians as their first source of information about clinical trials [24]. In another study conducted by the National Cancer Institute in the US, respondents identified a healthcare provider (73.3%) and patient organizations (13.5%) as their source of information about clinical trials [25].

In another US study, respondents asked “Which organizations listed below would you say have the greatest responsibility in educating the public about clinical trials?” most indicated physicians (44.0%) [26]. It is important to train and update the knowledge of specialists and GPs themselves, who will be able to explain to patients the general principles of clinical trials and their rights and responsibilities. Doctors can, in the first instance, provide information to their patients about the possibility of participating in clinical trials before they go to a research center. In addition, the activities of patient organizations should be supported as entities with, among other things, a real influence on the attitude of potential clinical trial participants. Patient organizations are important partners involved not only in initiating but also in monitoring and implementing new solutions in the healthcare system. Given the multiplicity and types of organizations operating in the health field, it seems natural to exploit their potential in the broader clinical trials market. Importantly, knowledge about clinical trials should be systematically raised among the organizations themselves [19].

Most participants (81.4%) agreed that participation in the clinical trial was completely voluntary. A very similar result (85.3%) was obtained in another study conducted in India [18]. Additionally, in our study, more than half of the respondents (53.7%) believed that hospitals participating in clinical trials provide better healthcare. In another study conducted in India, 67.2% of respondents also shared this view [18]. In a survey conducted in the US, 82.0% associated research with “good hospitals” [27]. In another study, 69.5% of respondents agreed with the statement that “Hospitals that participate in clinical research provide better healthcare” [14]. This is a positive sign, suggesting that there is some understanding of the benefits of involving hospitals in this type of research.

A total of 37.2% disagreed with the statement that the results of clinical trials are made available to the public. To the same statement, 34.5% of participants answered in the affirmative. The remaining 28.2% could not answer this question. Interestingly, in another study conducted in India, 28.0% of respondents were also unable to answer this question, while 37.0% agreed with the statement [18]. Knowledge on this topic of the availability of clinical trial results is crucial, as the transparency and accessibility of clinical trial results contribute to public trust in medical science and enable patients to make informed decisions about their treatment.

Our study found that 48.3% of participants said that it is possible to withdraw from a clinical trial at any time. A similar result was obtained in a study in Jordan published in 2020, where 49.2% of respondents also agreed with this statement [14]. A total of 30.3% of participants declared that clinical trials involving children should be conducted. In another study conducted in the USA, 81% of participants agreed that physicians should conduct research involving children [28]. In our study, most participants (64.5%) would allow their child to participate in a clinical trial as the only treatment option (no other treatment available). In another study conducted in the US, 53.4% of respondents thought it was appropriate for researchers to conduct trials involving children, and 46.9% said they would agree to allow their child to participate in a clinical trial [28].

The study also measured the strongest correlational relationships (i.e., those with |ρ| > 0.4 and *p* < 0.05) but showed no significant correlations. To the best of the authors’ knowledge, this is one of the most recent studies to assess knowledge and beliefs about clinical trials among Poles.

To reach the largest possible group of Polish patients, parallel educational activities on multiple levels may have the best effect. Further studies in different groups, i.e., clinical trial patients and investigators, are needed.

## 5. Limitations

The survey was carried out using the CAWI technique. Due to the characteristics of online surveys, there are some limitations [29,30]. First, the requirement for Internet access and computer literacy. However, according to a report by the Centre for Social Opinion Research in Poland, more than three-quarters of all adult Poles (77.0%) declare regular online presence (at least once a week) [31]. Secondly, there was a lack of an interviewer as a guide for respondents. However, it is worth noting that the questionnaire was accompanied by a short introduction that explained to respondents the purpose of the survey and how it would be conducted. Additionally, respondents do not have the opportunity to ask the interviewer questions and suggest prompts. This positively affects the reliability of the responses by keeping the same running conditions with each participant. Thirdly, loss of respondent attention can be a problem. Nevertheless, during the preparation of the questionnaire, we tried to narrow down and refine the questions in detail. Despite the effort to limit the number of questions and to make the questionnaire as clear as possible, there is a risk of respondents quickly reviewing the questionnaire. Although the questionnaire has been developed based on existing research and has undergone expert verification, as well as being tested on a limited group of non-medical people, these measures may not be sufficient. On the other hand, thanks to anonymity, the self-presentational impact on responses is reduced. Another limitation of the survey may be the response rate, which was 26.04%. For improved response rates in future surveys, sending reminders to those who did not respond to the first invitation can be considered.

## 6. Conclusions

The results of our survey illustrate the current level of awareness and knowledge about clinical trials in Poland. Poles’ knowledge of clinical trials is mainly based on commercial sources. Poles rate their knowledge of clinical trials as low and would like to learn more. To reach the largest possible group of Polish patients, parallel educational activities on multiple levels may have the best effect. Further studies in different groups, i.e., clinical trial patients and investigators, are needed. It seems appropriate to conduct an information campaign to raise public awareness of clinical trials. These activities should be carried out with the support of the media, healthcare institutions, and patient organizations, which will allow for a wide reach and effectiveness of the message. The implementation of these activities has the potential to significantly contribute to increasing Poles’ knowledge of clinical trials, which in the long run is crucial for the development of new, effective therapies.

## Figures and Tables

**Table 1:** Summary of methods.

Key Element	Details
Study Design	A cross-sectional study
Participants	Polish residents aged 18 or over
Data Collection Methods	The computer-assisted web interviewing (CAWI) method
Tools Used	A nationwide self-completion questionnaire
Statistical package used	R language environment

**Table 2:** Participants characteristics (*n* = 1079).

		*n*	(%)
Age (years), mean SD	44.96		
18–24		141	13.1
25–34		216	20.0
35–44		180	16.7
45–54		187	17.3
≥55		355	32.9
Sex			
Female		568	52.6
Male		511	47.4
Region			
Village		404	37.4
Small town ^1^		140	13.0
Medium town lower ^2^		96	8.9
Medium town upper ^3^		115	10.7
Large city lower ^4^		87	8.1
Large city upper ^5^		103	9.5
Big city ^6^		134	12.4
Education			
Primary or middle school		40	3.7
Basic		111	10.3
Secondary school		338	31.3
Post secondary school		111	10.3
Bachelor’s degree		96	8.9
Master’s degree		383	35.5
Voivodship			
Dolnośląskie		62	5.7
Kujawsko–pomorskie		59	5.5
Łódzkie		68	6.3
Lubelskie		71	6.6
Lubuskie		31	2.9
Małopolskie		97	9.0
Mazowieckie		155	14.4
Opolskie		24	2.2
Podkarpackie		63	5.8
Podlaskie		48	4.4
Pomorskie		55	5.1
Śląskie		136	12.6
Świętokrzyskie		34	3.2
Warminsko–mazurskie		30	2.8
Wielkopolskie		104	9.6
Zachodniopomorskie		42	3.9
Family status			
Single		333	30.9
Married		575	53.3
Divorced		116	10.8
Widowed		55	5.1

Population: up 20,000 inhabitants ^1^, 20,001–50,000 inhabitants ^2^, 50,001–100,000 inhabitants ^3^, 100,001–200,000 inhabitants ^4^, 200,001–500,000 inhabitants ^5^, more than 500 000 inhabitants ^6^.

**Table 3:** Adults in Poland’s awareness and knowledge about clinical trials (*n* = 1079).

	*n*	(%)
Awareness of clinical trials		
Yes	933	86.5
No	146	13.5
Self-reported knowledge about clinical		
Very strong	18	1.7
Strong	160	14.8
A little	466	43.2
Nothing at all	252	23.4
Hard to say	183	17.0
Willingness to learn more about clinical trials		
Definitely yes	238	22.7
Rather yes	567	52.5
Probably not	120	11.1
Definitely not	28	2.6
Hard to say	126	11.7
Have you ever taken part in a clinical trial?		
Yes	124	11.5
No	955	88.5

**Table 4:** Beliefs and attitudes about clinical trials among adults in Poland (*n* = 1079).

	Definitely Yes		Rather Yes		Probably Not		Definitely Not		Hard to Say	
	*n*	(%)	*n*	(%)	*n*	(%)	*n*	%	*n*	(%)
Participation in a clinical trial is entirely voluntary	392	36.3	487	45.1	39	3.6	9	0.8	152	14.1
Hospitals participating in clinical trials provide better healthcare	131	12.1	449	41.6	109	10.1	24	2.2	366	33.9
The results of clinical trials are made available to the public	52	4.8	321	29.7	313	29.0	89	8.2	304	28.2
You can withdraw from a clinical trial at any time	126	11.7	395	36.6	190	17.6	28	2.6	340	31.5
The clinical trial patient is insured	179	16.6	459	42.5	110	10.2	13	1.2	318	29.5
All clinical trial patients receive an investigational medicinal product	59	5.5	259	24.0	243	22.5	228	21.1	290	26.9
Clinical trials should be conducted with children	70	6.5	257	23.8	226	20.9	160	14.8	366	33.9
I would allow my child to participate in a clinical trial as the only therapeutic option (there is no other treatment)	237	22.0	459	42.5	74	6.9	66	6.1	243	22.5

## Data Availability

The datasets used and/or analyzed during the current study are available from the corresponding author upon reasonable request.
